# Glycosphingolipids in Cardiovascular Disease: Insights from Molecular Mechanisms and Heart Failure Models

**DOI:** 10.3390/biom14101265

**Published:** 2024-10-08

**Authors:** Sarah Huang, Karima Abutaleb, Sumita Mishra

**Affiliations:** 1 Department of Medicine, Division of Cardiology, The Johns Hopkins University School of Medicine, Baltimore, MD 21218, USA; 2 Department of Surgery, Virginia Tech Carilion School of Medicine, Roanoke, VA 24061, USA; 3 Center for Exercise Medicine Research, Fralin Biomedical Research Institute, Virginia Tech, Roanoke, VA 24061, USA; 4 Center for Vascular and Heart Research, Fralin Biomedical Research Institute, Virginia Tech, Roanoke, VA 24061, USA; 5 Department of Human Nutrition, Foods, and Exercise, College of Life Sciences, Virginia Tech, Roanoke, VA 24061, USA

**Keywords:** glycosphingolipids, heart failure, HFrEF, HFpEF, ceramide

## Abstract

This review explores the crucial role of glycosphingolipids (GSLs) in the context of cardiovascular diseases (CVDs), focusing on their biosynthesis, metabolic pathways, and implications for clinical outcomes. GSLs are pivotal in regulating a myriad of cellular functions that are essential for heart health and disease progression. Highlighting findings from both human cohorts and animal models, this review emphasizes the potential of GSLs as biomarkers and therapeutic targets. We advocate for more detailed mechanistic studies to deepen our understanding of GSL functions in cardiovascular health, which could lead to innovative strategies for diagnosis, treatment, and personalized medicine in cardiovascular care.

## 1. Introduction

Glycosphingolipids (GSLs) are a diverse group of bioactive molecules essential for cell membrane structure and function across various organisms, from bacteria to humans [1]. Each GSL comprises a hydrophobic ceramide backbone made up of a fatty acid and a sphingoid base and a hydrophilic carbohydrate head group, the composition of which ranges from a simple sugar to complex oligosaccharides. The structural variety is significant, featuring over 400 glycan structures in vertebrates, varying in length from one to twenty sugar residues and incorporating up to twelve different sugar types [2]. Additionally, the ceramide backbone introduces further diversity, with more than 200 distinct species identified in mammalian cells, differentiated by variations in chain length, double bonds, and hydroxyl groups [2].

GSLs are broadly categorized into seven main types based on their glycan components—ganglio-, globo-, isoglobo-, lacto-, and neolacto-series prevalent in vertebrates and mollu- and arthro-series primarily found in mollusks and arthropods (Table 1) [1]. These GSLs are also classified by charge into neutral, sialylated, or sulfated forms, where neutral GSLs contain uncharged sugars and sialylated GSLs include negatively charged sialic acid residues [3].

GSLs are integral in mediating a variety of biological processes, including cell division, differentiation, and signaling [4,5]. Positioned strategically within plasma membranes, GSLs facilitate critical interactions that regulate cell adhesion, motility, and communication [6,7,8]. These interactions are central to signaling pathways that control cell proliferation, migration, autophagy, apoptosis, and mitochondrial function [9].

In the bloodstream, GSLs notably associate with low-density lipoproteins (LDLs), playing a pivotal role in lipid transport and metabolism [10]. This association is crucial for receptor-dependent endocytic pathways that are fundamental to lipid homeostasis and cellular signaling. The dynamic interactions of GSLs with various cellular components significantly impact angiogenesis and inflammation, which are vital in both disease progression and the development of therapeutic strategies [11].

At the cellular level, GSLs influence essential membrane proteins such as growth factor receptors (GFRs) and integrins. For example, the GM3 ganglioside can inhibit the autophosphorylation of the epidermal growth factor receptor (EGFR), thereby disrupting signaling pathways crucial for cancer progression [12]. GSLs also play roles in cell adhesion through interactions with galectin-3 and E-selectin, which are crucial for cell tethering and migration. Furthermore, they regulate cell motility by interacting with tetraspanins and integrins within glycosynapses, specialized domains that contain clusters of GSLs and modulate cellular responses to external stimuli [13].

Alterations in GSL metabolism are implicated in various diseases, including cancer, where different GSL metabolites can have distinct and sometimes opposing roles in tumor growth and invasiveness [14]. They are also involved in neurodegenerative diseases like Alzheimer’s and Parkinson’s diseases, where they influence protein aggregation [15]. Additionally, in cardiovascular disorders, GSLs are linked to atherosclerosis and heart failure [5,16,17]. The therapeutic targeting of GSL biosynthesis pathways highlights their potential as universal targets in oncology, neurology, and cardiovascular medicine.

## 2. Biosynthesis and Metabolism of Glycosphingolipids

### 2.1. GSL Synthesis

GSL synthesis initiates in the endoplasmic reticulum (ER), where serine palmitoyltransferase (SPT), a key enzyme located in the ER membrane, catalyzes the condensation of L-serine and palmitoyl-CoA. This reaction forms 3-ketodihydrosphingosine, which is subsequently reduced to sphinganine by 3-ketodihydrosphingosine reductase (KDHR), a reaction dependent on nicotinamide adenine dinucleotide phosphate (NADPH). Following sphinganine formation, it is acylated by ceramide synthase (CerS) with fatty acyl-CoA to form dihydroceramide. This step is pivotal, as different CerS isoforms (CerS1–6) with specificity for various acyl chain lengths direct the synthesis of distinct ceramides, influencing diverse cellular functions from apoptosis to cell survival [18,19]. These dihydroceramides are then desaturated by dihydroceramide desaturase to form ceramides, which are integral components of complex GSLs [20].

In parallel, the salvage or catabolic pathway plays a crucial role in GSL metabolism, in which sphingolipids are recycled through hydrolysis at the plasma membrane or in lysosomes. Here, complex GSLs are first reverted to ceramide, which serves as a substrate for the generation of new sphingolipids or for catabolism, into sphingosine and, subsequently, into sphingosine-1-phosphate (S1P), a potent signaling molecule [21].

### 2.2. Transport and Glycoslyation

**Figure 1:**
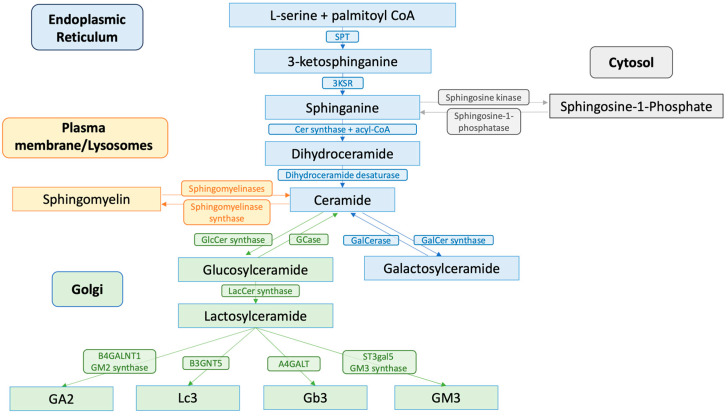
Biosynthesis and catabolism of glycosphingolipids. SPT = serine palmitoyltransferase; 3KSR = 3-ketosphingosine reductase; Cer synthase = ceramide synthase; GlcCer synthase = glucosylceramide synthase; GCase = glucocerebrosidase; GalCerase = galactosylceraimdase; GalCer synthase = galactosylceramide synthase; LacCer synthase = lactosylceramide synthase; B4GALNT1 = β4-N-acetylgalactaminyltransferase 1; B3GNT5 = β3-N-acetylglucosaminyltransferase 5; A4-GALT = α4-galactosyltransferase; ST3GAL5 = α3-sialyltransferase 5.

Once synthesized, ceramides are transported to the Golgi apparatus. This transfer is facilitated by the ceramide transfer protein (CERT), which shuttles ceramide from the ER to the trans-Golgi network for further processing into GSLs like glucosylceramide (GlcCer) and lactosylceramide (LacCer). These molecules are foundational for the synthesis of higher-order GSLs such as gangliosides and globosides [15,22]. Glycosylation reactions in the Golgi extend the ceramide backbone into diverse GSL structures, which are then transported to the plasma membrane, contributing to the lipid raft architecture and affecting signal transduction processes (Figure 1).

### 2.3. GSL Degradation

GSL degradation involves endocytosis of membrane-bound GSLs and transport to endosomal vesicles, followed by the sequential removal of sugar residues from the non-reducing end, a process that is catalyzed by glycosyl hydrolases (GHs). GH activity is often facilitated by sphingolipid activator proteins, or saposins (SAPs), which provide the water-soluble hydrolases access to their membrane-embedded substrates. Upon cleavage in the lysosome, precursor protein prosaposin gives rise to four SAP variants (SapA–D), which each have affinities for specific GSLs and function specifically with their associated hydrolase [23]. Following the degradation of the glycan chains, ceramidases hydrolyze ceramide, resulting in sphingosine and fatty acid [24]. There are three types of ceramidases, characterized by their optimal pH ranges, namely acid, neutral, and alkaline ceramidase. Acid ceramidase contains a mannose-6-phosphate tag that localizes it to the lysosome, where it hydrolyzes ceramides from the endosomal membrane system from the plasma membrane. Its optimal pH for enzymatic activity is between 4.5 and 5 [25]. Neutral ceramidase localizes primarily to the plasma membrane but can also be found in the Golgi and mitochondria and is mainly expressed in the small intestine and colon. The enzyme regulates sphingosine and S1P production across the plasma membrane [26]. Finally, there are three alkaline ceramidases. ACER1 localizes to the ER and is primarily expressed in the skin in epidermal keratinocytes. ACER2 localizes to the Golgi and may play a role in DNA damage and the MAPK signaling pathway. ACER3 localizes to both the ER and Golgi and specifically hydrolyzes ceramides carrying unsaturated long acyl chains [27].

## 3. Spatial Dynamics of Glycosphingolipid Signaling in Cardiac Cellular Compartments

### 3.1. GSLs in Membrane Microdomains and Mitochondria-Associated ER Membranes (MAMs)

GSLs play vital roles in the structure of plasma membranes, with varying concentrations and compositions of different GSLs leading to changes in various membrane properties. For example, an increase of GlcCer within fluid POPC (1-palmitoyl-2-oleoyl-sn-glycero-3-phosphocholine, a commonly used lipid model for biophysical experiments) membranes was shown to drive the formation of gel domains, and even higher levels led to the formation of crystal-like structures. In vesicles, high concentrations of GlcCer led to the protrusion of tubule-like structures from the vesicles, which may be driven by changes in membrane curvature and lipid phase changes [28]. Within the plasma membrane, GSLs are typically found in lipid rafts, which are microdomains enriched in sphingolipids, cholesterol, and specific proteins. Due to their unique properties, including their geometry and ability to form hydrogen bonds, GSLs contribute to the formation and stabilization of these lipid rafts [29]. Lipid rafts serve as signaling platforms, enhancing intracellular communication by clustering signaling molecules and substrates [3]. Additionally, GSLs are enriched in extracellular vesicles (EVs), which are involved in cell-to-cell signaling. Lipid-enriched EVs can induce apoptosis and activate immune responses, affecting cellular processes [30].

In the cardiovascular system, lipid rafts regulate signaling pathways, controlling heart rate, vascular tone, and endothelial function [31]. GSLs within lipid rafts modulate the activity of ion channels and receptors, such as G-protein-coupled receptors and integrins, which are crucial for the physiological responses of the heart and blood vessels. Alterations in GSL composition can disrupt raft integrity and functionality, impairing signal transduction and contributing to cardiovascular diseases such as hypertrophy, atherosclerosis, hypertension, and cardiac arrhythmias [3,5,16,17,29]. Another key microdomain involving GSLs is the mitochondria-associated ER membrane (MAM), a contact site between the ER and mitochondria that regulates Ca^2+^ dynamics, lipid synthesis, apoptosis, and energy metabolism [32]. Gangliosides, a subclass of GSLs, are particularly important in MAMs. For instance, the ganglioside GD3 has been implicated in inducing autophagy via interactions with calnexin, a resident protein of MAMs [33]. Similarly, GM1 binds Ca^2+^ and can disrupt Ca^2+^ buffering in MAMs, particularly under disease conditions. Accumulation of GM1 within MAMS has been shown to disturb mitochondrial dysfunction, leading to depolarization, membrane permeabilization, and apoptosis [32]. In mouse models with β-galactosidase (β-gal) mutations, defective lysosomal degradation of GM1 results in its accumulation in MAMs, which can lead to the clustering of IP3R-1 (an intracellular calcium channel) and the formation of a Ca^2+^ mega pore. This further increases mitochondrial Ca^2+^ influx, disrupting the mitochondrial membrane potential [34].

Caveolae, invaginated lipid microdomains rich in GSLs, are significant in cardiovascular health. The loss of the function of caveolin, the major membrane proteins of caveolae, is linked to vascular disorders and hypertension, possibly due to its role in endothelial nitric oxide synthase (eNOS) signaling. eNOS, which is localized in caveolae, is associated with caveolin-1 (Cav1), inhibiting eNOS activity and nitric oxide (NO) production [35]. Increased Cav1 expression is observed in patients with insulin resistance and type 2 diabetes [36], while Cav1 knockout mice exhibit increased systemic NO and dilated ventricles [37]. Reduced NO production is associated with atherosclerosis, the pathological basis of many cardiovascular disorders.

### 3.2. GSLs in Mitochondrial Function and Dynamics

**Figure 2:**
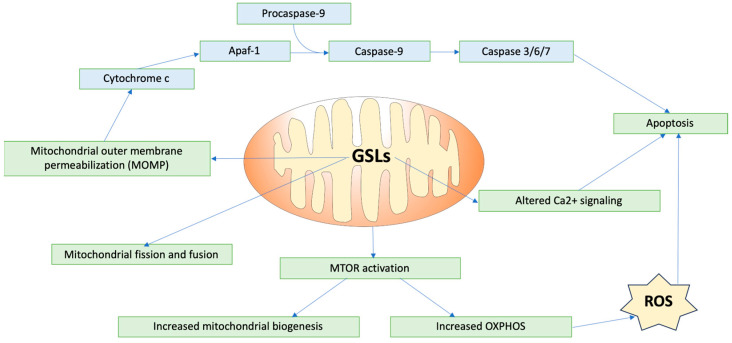
Effects of GSLs on mitochondrial processes. ROS = reactive oxygen species; OXPHOS = oxidative phosphorylation; mTOR = mechanistic target of rapamycin, Apaf-1 = apoptotic protease activating factor 1.

GSLs play critical roles in modulating mitochondrial dynamics and signaling, impacting cellular processes like energy production and apoptosis (Figure 2). One key area of regulation that GSLs are involved in is mitochondrial membrane permeabilization, a critical event in apoptosis. Ceramide, a fundamental GSL, forms channels within mitochondrial membranes, leading to mitochondrial outer-membrane permeabilization [19]. Ganglioside GD3 has also been shown to suppress proton-pumping activity and/or increase small conductance channels, inducing a gradual depolarization of the inner mitochondrial membrane [38]. This permeabilization and depolarization facilitate the release of cytochrome c, triggering caspase activation and cell death mediated through interactions with B-cell lymphoma-2 (BCL-2) family proteins [39]. In the cardiac system, this relationship is especially important, considering the critical role of mitochondrial function to cardiomyocyte performance, and dysregulation of this pathway may lead to adverse cardiac outcomes.

Furthermore, GSLs are integral to the regulation of mitochondrial fission and fusion dynamics, processes vital for the maintenance of mitochondrial integrity and function. Mitochondrial dynamics control the transport, size, morphology, and turnover of mitochondria, directly affecting cell viability and ATP production, an important aspect for cells such as cardiomyocytes, which have high metabolic demand. Disruption of this fission/fusion balance can lead to alterations in mitochondrial function, which, in turn, are associated with several cardiac diseases [40]. GD3, a ganglioside present in MAMs, has also been shown to interact with proteins involved in initiating autophagy, such as WIPI1 and AMBRA1 [33]. Furthermore, following CD95/Fas triggering (activation of death receptors that trigger apoptosis), GD3 has been shown to localize to mitochondrial fission regions, along with other fission-involved proteins such as dynamin-like protein 1 (DLP1) and human Fis1 protein (hFis1) [41]. This relationship may also occur with less complex GSLs such as glucosylceramide and lactosylceramide, resulting in increased synthesis of mitochondrial fusion and fission proteins, indicating increased mitochondrial turnover [42]. Ceramide also influences the recruitment of proteins like Drp1 to mitochondrial raft-like domains, promoting mitochondrial fission [43].

Additionally, GSLs play a role in regulating mitochondrial calcium retention capacity and energy production. Mitochondrial Ca^2+^ uptake is important in regulating contractile activity through the modulation of cellular metabolism and energy. In the hearts of mice with type-1 diabetes, lactosylceramide was shown to inhibit the electron transport chain and decrease calcium retention capacity. Specifically, LacCer suppressed state 3 respiration (characterized by maximal mitochondrial oxygen consumption after the addition of ADP) by targeting complexes I, II, and IV in the electron transport chain (ETC). In terms of calcium retention, the addition of LacCer to mitochondria led to the release of Ca^2+^ from the mitochondria, causing mitochondrial dysfunction. A possible mechanism for this release could be a LacCer-induced increase in Ca^2+^ sensitivity of the mitochondrial permeability transition pore (mPTP) [44]. GD3 also impacts the protein complexes of the ETC. Dysregulation of GD3 synthesis and metabolism leads to ROS production, activating the PI3K/Akt/mTOR pathway, which regulates mitochondrial biogenesis and oxidative phosphorylation [42].

### 3.3. GSLs in Cellular Signaling Pathways

Sphingolipids such as ceramide and sphingosine-1-phosphate (S1P) play pivotal roles in cardiac function and disease by modulating a vast array of cellular processes. Ceramide, for instance, is known to escalate cytosolic Ca^2+^ levels, thereby influencing cardiac hypertrophy or contractile function during ischemia–reperfusion (IR) events. It activates key signaling pathways such as p38 MAPK and PKC, which concurrently inhibit AKT and JNK pathways, thereby triggering pro-apoptotic BAX signaling. This dual role of ceramide in promoting cellular adaptations to stress while inducing detrimental effects highlights its complex involvement in cardiomyocyte physiology [45].

In contrast, S1P offers cardioprotective effects through its intricate signaling mechanisms. It is produced under the influence of hypoxic transcription factors like HIF1α and HIF2α or pro-inflammatory mediators such as TNF-α, activating a cascade of pro-survival pathways including AKT, PI3K, Pak1, and Rac. These pathways notably decrease infarct size and promote endothelial cell migration and angiogenesis, enhancing the viability of cardiomyocytes in both in vitro and in vivo IR models [46]. Additionally, S1P interactions with its specific receptors activate pathways like Pak1 and protein phosphatase 2A (PP2A), which are essential for cardiac protection in conditions such as ischemic and Takotsubo cardiomyopathy [47]. S1P also plays a critical systemic role by mediating renin release and stimulating aldosterone secretion within the renin–angiotensin–aldosterone system (RAAS), which is crucial for the maintenance electrolyte and fluid balance, thereby stabilizing blood pressure in conditions like hypertrophic cardiomyopathy [48].

Furthermore, GM1 ganglioside, through its oligosaccharide chain, independently interacts with plasma membrane receptors such as TrkA, the receptor for the nerve growth factor (NGF). This interaction initiates the MAPK/ERK signaling pathway, promoting cellular differentiation and survival, which are crucial in cardiac stress responses and repair mechanisms [49]. This function of GM1 illustrates the non-structural, signaling-mediated roles of GSL oligosaccharides in cellular modulation, emphasizing their potential in therapeutic strategies targeting various cardiomyopathies and cardiovascular dysfunctions.

## 4. Glycosphingolipids in Cardiovascular Disease Pathogenesis

Heart disease remains the leading cause of mortality in the United States, accounting for one in every five deaths in 2021 and representing a substantial portion of healthcare expenditures [50,51]. Cardiovascular disorders, including hypertension, coronary artery disease, cardiomyopathy, atherosclerosis, and heart failure, are influenced by diabetes, obesity, poor diet, physical inactivity, and excessive alcohol use [51]. GSLs have been linked to the progression of these cardiovascular conditions [5,16,39,50,52,53].

### 4.1. Atherosclerosis

**Figure 3:**
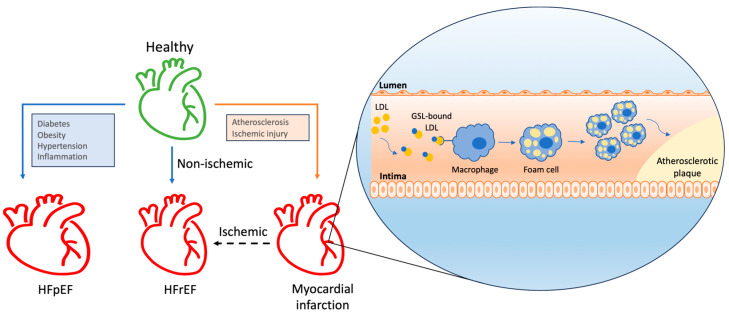
GSL involvement in atherosclerosis, leading to heart failure. HFpEF = heart failure with preserved ejection fraction; HFrEF = heart failure with reserved ejection fraction; LDL = low-density lipoprotein.

The role of GSLs in modulating the inflammatory response within the cardiovascular system contributes to atherosclerosis, a crucial factor in the development of heart failure (Figure 3). While the complete mechanisms of atherosclerosis progression remain to be fully elucidated, lipoprotein retention in the vascular intima is a known initiating factor. Accumulated lipoproteins (predominantly LDLs) prompt endothelial cells to recruit monocytes, which differentiate into macrophages and, subsequently, into foam cells [53]. GSLs have been observed accumulating in the aortic walls of patients with atherosclerosis; tissue samples from these patients, showing fatty streaks and atherosclerotic plaques, revealed increased levels of glucosylceramide, lactosylceramide, and GM3. Furthermore, an upsurge in gangliosides GD3 and GD1a was detected in the media beneath the atherosclerotic lesions [54]. The pro-atherosclerotic mechanism likely involves GSLs binding LDLs. Gangliosides have been demonstrated to bind LDLs, leading to alterations in LDL structure by inducing conformational changes in apoB molecules on the LDL surface, promoting LDL particle aggregation and enhanced uptake by macrophages [10]. Our previous studies have also shown that inhibiting glycosphingolipid synthesis reduces atherosclerosis and arterial stiffness in apolipoprotein E (*−*/*−*) mice and rabbits. Feeding these animals a Western diet significantly increased aortic pulse-wave velocity, intima-media thickening, levels of oxidized low-density lipoprotein, Ca^2+^ deposits, and glucosylceramide and lactosylceramide synthase activity. These effects were dose-dependently reduced by GSL synthesis inhibitor D-PDMP. In the liver, D-PDMP lowered cholesterol and triglyceride levels by enhancing the expression of SREBP2, the LDL receptor, HMGCo-A reductase, and cholesterol efflux genes such as ABCG5 and ABCG8. D-PDMP also affected VLDL catabolism by upregulating the expression of lipoprotein lipase and the VLDL receptor. Rabbits fed a Western diet for 90 days exhibited extensive atherosclerosis, accompanied by a 17.5-fold increase in total cholesterol levels and a 3-fold increase in lactosylceramide levels, all of which were completely prevented by D-PDMP administration [17]. However, a study by Glaros et al. indicated that inhibiting GSL synthesis through the inhibition of glucosylceramide synthesis did not impact the atherosclerotic lesion area in mice, suggesting a complex role for GSLs in atherosclerosis [55].

S1P, a GSL precursor, has been demonstrated to affect atherosclerosis both positively and negatively. By inhibiting macrophage apoptosis and endothelial inflammation via the PI3K/Akt pathway, S1P’s interaction with S1PR1 may have anti-atherosclerotic effects. Conversely, its interaction with S1PR2/3 may inhibit the proliferation and migration of smooth muscle cells by interacting with Rac protein, thereby delaying or inhibiting neointima formation. However, S1P has also been shown to activate pro-inflammatory and endothelial factors, including ICAM-1, VCAM-1, and IL-6, potentially exacerbating atherosclerosis [56]. Ceramides also play a significant role in atherosclerosis development. They accumulate in atherosclerotic plaques and may initiate plaque formation. The inhibition of SPT reduces plasma sphingolipid concentrations, including ceramides, leading to smaller atherosclerotic lesions in the aortas of atherosclerotic mice [53]. Endothelial dysfunction, a precursor to atherosclerosis, can be induced by ROS generation and reduced nitric oxide (NO) availability. Ceramides have been linked to elevated ROS levels, and conversely, ROS can stimulate ceramide synthesis. Ceramides also activate ROS-generating enzymes such as NADPH oxidase, uncoupled eNOS, and xanthine oxidase following stimulation by pro-inflammatory cytokines such as TNF. Other critical processes in atherosclerotic lesion development, such as LDL aggregation and monocyte recruitment, have been associated with ceramide levels [57].

### 4.2. GSLs in Angiogenesis and Inflammation

GSLs may influence cardiac remodeling through their role in angiogenesis. Globo H ceramide (GHCer), a prevalent cancer-associated GSL, exhibits proangiogenic activity. GHCer induced migration, tube formation, and intracellular calcium mobilization in human umbilical vein endothelial cells (HUVECs). Additionally, subcutaneous injection of GHCer plugs into mice increased blood vessel formation. These effects are linked to the co-localization of GHCer with translin-associated factor X (TRAX), wherein GHCer competes with phospholipase C β1 (PLCb1) for binding to TRAX. GHCer effectively inhibits TRAX, activating PLCb1 and leading to calcium mobilization and angiogenesis [11].

GSL-enriched microdomains modulate immune receptor signaling across various immune cell types, affecting both innate and adaptive responses. Lactosylceramide (LacCer) is highly expressed in human neutrophils and binds various pathogenic microorganisms. LacCer-enriched lipid rafts contain Src family tyrosine kinase Lyn and mediate the generation of proinflammatory cytokines and superoxide in neutrophils [58]. Changes in the expression of GSL metabolism genes are associated with fibroblast differentiation in keloids, pathological scars characterized by inflammation and excess collagen [59]. Specifically, within the cardiovascular system in patients with myocardial infarction, sphingomyelin, ceramide, and glucosylceramide have been shown to be positively correlated with high-sensitivity C-reactive protein, a marker of acute inflammation [60]. Cer and sphingomyelin have also been shown to modulate cellular responses to cytokines. For example, C2 and C6 both potentiated the induction of C-reactive protein (CRP) and serum amyloid A (SAA) in Hep3B cells [61].

In macrophages, GM3-enriched microdomains interact with insulin receptors to inhibit insulin signaling, impacting metabolic responses [62]. Similarly, in adipocytes, these interactions suppress the autophosphorylation of EGF receptors, thereby modulating growth signaling pathways [63]. Furthermore, GSL microdomains regulate toll-like receptor (TLR) functions in dendritic cells and macrophages; for example, the interaction of glucosylceramide with TLR4 influences its orientation and signaling response to lipopolysaccharide, enhancing immune activation via MyD88 adapter proteins [64]. This mechanistic role extends to T-cell regulation, where GM1 microdomains interact with neurotrophin receptors like TrkA, influencing T-cell differentiation and survival [65]. On the other hand, proteins involved in GSL metabolism, such as S1PR1, may protect against myocardial injury by soothing inflammatory responses, stimulating the proliferation of repair macrophages, and inhibiting myocardial fibrosis through the Akt/eNOS-dependent pathway [56]. These specific interactions across different immune cells underscore the sophisticated regulatory capabilities of GSL-enriched microdomains in orchestrating immune responses and maintaining cellular homeostasis.

### 4.3. Hypertrophy and Heart Failure

Cardiac hypertrophy, which is defined by an increase in cardiomyocyte size in response to ventricular wall stress and pressure overload, initially serves as a compensatory mechanism to enhance contractility by increasing the number of sarcomere units and reducing left ventricular wall stress, thereby maintaining cardiac efficiency. However, this adaptive hypertrophy can progress to pathological hypertrophy, leading to heart failure. Heart failure, which is characterized by the heart’s inability to pump blood effectively, is divided into two major types, namely heart failure with reduced ejection fraction (HFrEF) and heart failure with preserved ejection fraction (HFpEF) [50].

#### 4.3.1. GSL Insights from HFrEF

HFrEF is characterized by dilated ventricles, decreased contractility, and reduced ejection fraction. This form of heart failure can arise from ischemic causes, such as ischemic injury, or non-ischemic causes. Disrupted GSL metabolism has been linked to the pathogenesis of heart failure. Specifically, serine palmitoyltransferase (SPT), which catalyzes the condensation of serine and palmitoyl-CoA, is crucial for cardiovascular health. In mice, the expression of SPTLC3, a subunit that broadens the substrate specificity of SPT, enables the generation of d16-derived sphingolipids from myristoyl-CoA, promoting cell death and the cleavage of poly (ADP-ribose) in cardiomyocytes [66]. Furthermore, ER membrane protein Nogo-A, which inhibits SPT, is upregulated in cardiomyocytes following pressure overload, thereby preventing ceramide accumulation and protecting the heart from failure [67]. In other mouse models of heart failure, altered lipid content within the heart is associated with the onset and progression of heart failure. In mice subjected to transverse aortic constriction, early sphingolipid shifts include an increase in dihydrosphingosine, with subsequent decreases in erythro-sphingosylphosphorylcholine and stearoyl sphingomyelin as heart failure progresses [68]. Additionally, in mice with cardiac-specific angiotensin II overexpression (TG1306/R1), studies highlight a distinct sphingolipid profile characterized by exacerbated heart size, systolic dysfunction, and cardiac fibrosis, accompanied by significant changes in ceramide species [69]. This finding correlates with emerging evidence that suggests a relationship between the fatty acyl chain length of ceramides and their impact on cardiac function. A higher ratio of C16:0 to C24:0 ceramides is associated with worse left ventricular dysfunction, decreased left atrial function, increased left atrial size, overall mortality, and increased incidence of heart failure [39].

Oxidative stress plays a critical role in cardiomyocyte death and fibrosis, leading to pathological hypertrophy and heart failure. Our studies have shown that GSLs such as lactosylceramide (LacCer) can generate reactive oxygen species (ROS) and activate ERK-1/p44 MAPK in cardiomyocytes, ultimately leading to hypertrophy [5]. Treatment with GSL synthesis inhibitor D-PDMP prevented cardiac hypertrophy in mice fed a high-fat, high-cholesterol diet and reduced the expression of BNP and ANP, biomarkers of cardiac hypertrophy. D-PDMP also decreased GSL mass in heart tissue by inhibiting glycosyltransferase activity [16].

Furthermore, UGCG (GlcCer synthase) and B4GalT5 (the enzyme converting glucosylceramides to lactosylceramides) may form a complex regulating mitochondrial oxidative stress. The inhibition of UGCG in cardiomyocytes led to reduced mitochondrial ROS levels, while its overexpression increased ROS levels. Inhibition of B4GalT5 improved mitochondrial ROS levels, even after UGCG overexpression, suggesting an involvement mediated by ERK signaling, i.e., the activation of the UGCG-B4GalT5 axis was correlated with ERK activation, and the downregulation of UGCG reduced ERK activation and myocardial hypertrophy [70]. Elevated levels of glucosylceramide (GlcCer) have been observed in heart tissue in patients with reduced ejection fraction (EF) following an ischemic event compared to those with normal heart function. This trend was also evident in mice subjected to induced myocardial infarction, indicating a potential role for GSLs in heart remodeling [71]. These findings are supported by studies conducted using mouse models with pressure-overloaded hearts, where UGCG was upregulated. The downregulation of UGCG ameliorated heart hypertrophy, as evidenced by reduced ventricular wall hypertrophy, lower heart weight-to-body weight ratios, decreased left ventricular weight-to-body weight ratios, reduced heart weight-to-tibial length ratios, and decreased cardiac fibrosis. Conversely, the overexpression of UGCG exacerbated heart hypertrophy following transverse aortic constriction (TAC) modeling [64]. Another study found an inverse relationship between specific ceramides (glycosyl ceramide, glycosyl–N–nervonoyl–sphingosine, lactosyl–N–nervonoyl–sphingosine, and lactosyl–N–palmitoyl–sphingosine) and the left ventricular mass-to-volume ratio, a measure of concentric cardiac remodeling [72].

**Figure 4:**
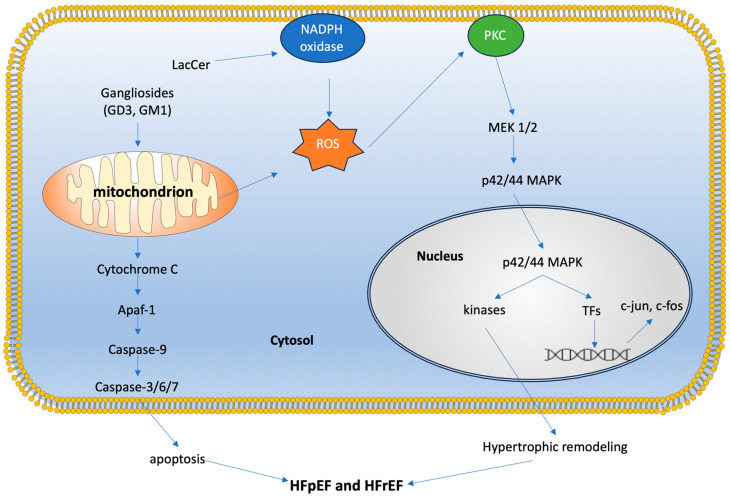
LacCer activates NADPH oxidase to generate reactive oxygen species, leading to protein kinase-C activation, p44-mitogen-activated protein kinase phosphorylation, and nuclear factor c-fos and c-jun expression, as well as cardiac hypertrophy. Gangliosides GD3 and GM1 interact with mitochondrial elements to induce caspase activation and set off a cascade of events leading to apoptosis. LacCer = lactosylceramide; NADPH = nicotinamide adenine dinucleotide phosphate; ROS = reactive oxygen species; PKC = protein kinase C; MEK = mitogen-activated protein kinase kinase (aka MAP2K); p42/44 MAPK = mitogen-activated protein kinases (aka Erk2 and Erk1); TFs = transcription factors; Apaf-1 = apoptotic protease-activating factor 1; HFpEF = heart failure with preserved ejection fraction; HFrEF = heart failure with reduced ejection fraction.

These findings underscore the significance of increased GSL levels in contributing to oxidative stress and cardiac hypertrophy, ultimately leading to heart failure (Figure 4).

#### 4.3.2. GSL Insights from Cardiometabolic Diseases and HFpEF

Heart failure with preserved ejection fraction (HFpEF) is a growing concern due to its increasing prevalence and strong association with metabolic disturbances. Obesity and type 2 diabetes mellitus are major drivers of HFpEF pathophysiology, primarily due to their significant impact on metabolic regulation. GSLs are increasingly recognized for their role in metabolic dysregulation, including insulin resistance and lipid metabolism, which contribute to myocardial remodeling and vascular dysfunction, which are characteristic of HFpEF [50].

A hallmark of cardiometabolic diseases is excess lipid deposition in non-adipose tissues, including the heart. Normally, fatty acids are metabolized via beta-oxidation to produce ATP, with excess free fatty acids stored as inert triglycerides within cells. However, when these metabolic pathways become saturated, bioactive lipids such as complex sphingolipids and ceramides accumulate in non-adipose tissues, including blood vessels and cardiac tissue. This lipid accumulation, or lipotoxicity, contributes to the development of cardiometabolic disorders [5,16,53]. For example, studies in Dahl salt-sensitive (Dahl/SS rats), a model for HFpEF due to sodium sensitivity, showed pre-heart failure increases in N-palmitoyl-sphingosine (C16:0) and glycosyl-N-stearoyl-sphingosine (C18:0), pointing to their potential as early biomarkers of heart failure [73].

GSLs are particularly involved in lipotoxic cardiomyopathy, acting as second messengers in pathways that lead to disease phenotypes such as proliferation, adhesion, migration, autophagy, apoptosis, and mitochondrial dysfunction [42] (Figure 4). Specific GSLs participate in signaling pathways that regulate cellular energy utilization, lipid metabolism, and inflammatory responses, all of which are crucial in HFpEF pathology. The overaccumulation of certain GSLs in cardiac cells disrupts normal metabolic processes, leading to inefficient energy use and increased oxidative stress, which are hallmarks of HFpEF. Additionally, GSLs influence the inflammatory environment within the myocardium, affecting fibrotic processes that contribute to heart muscle stiffening [52]. Modulating GSL pathways may mitigate metabolic disturbances and inflammatory responses, thereby improving cardiac function and patient outcomes.

Obesity is linked to an altered lipidome and is a common comorbidity in HFpEF. A study investigating the effects of weight loss in women with obesity and HFpEF demonstrated that gastric bypass surgery led to significant cardiac improvements, including reduced left ventricular mass and relative wall thickness and enhanced left ventricular relaxation [74]. Moreover, weight loss was associated with decreased plasma levels of several sphingolipids (C23:0, C24:0, SM 14:1, SM14:0, SM20:0, SM22:1, SM21:0, and SM22:0), while several others had increased levels (SM18:0, SM24:1, and SM24:2) [74]. These findings underscore the pivotal role of GSLs in cardiometabolic diseases.

#### 4.3.3. Diabetes

In diabetes, the dysregulation of sphingolipid metabolism, particularly ceramides, plays a pivotal role in modulating cellular signaling pathways that are crucial for the maintenance of metabolic homeostasis. Ceramides induce mitochondrial dysfunction and elevate oxidative stress, which are implicated in the disruption of insulin signaling pathways. Specifically, ceramides facilitate the inhibition of AKT translocation and the activation of conventional protein kinase C (PKC) isoforms, mechanisms that critically attenuate insulin-mediated glucose uptake [75]. Furthermore, ceramides trigger the activation of the mitogen-activated protein kinase (MAPK) pathway via p38, which concurrently inhibits AKT and activates c-Jun N-terminal kinase (JNK). This sequence of events promotes the activation of the pro-apoptotic B-cell lymphoma 2-associated X protein (BAX), leading to apoptosis and contributing to myocardial hypertrophy in diabetic cardiomyopathy [76]. Diacylglycerol acyltransferase (DGAT1), the enzyme that catalyzes the final step in triglyceride synthesis, has been shown to affect insulin resistance [77], and inhibition of this enzyme may be beneficial in patients with type 2 diabetes. However, humans with severe heart failure have notably reduced levels of DGAT1 mRNA in the heart. Indeed, cardiomyocyte-specific DGAT1 knockout mice exhibit severe increases in ceramide and diacylglycerol, as well as cardiomyopathy, linking aberrant metabolism of these lipids to diabetes and heart failure [78]. Additionally, glycosphingolipid glucosylceramide (GlcCer) exerts effects independent of ceramide, impairing insulin signaling through yet-to-be-elucidated mechanisms, thereby exacerbating insulin resistance [79]. This detailed mechanistic insight underscores the complex interplay between sphingolipid metabolism and key regulatory pathways affecting cellular function in diabetes.

### 4.4. Fabry’s Disease

Fabry’s disease (FD) is an X-linked genetic disorder caused by mutations in the GLA gene, which encodes the α-galactosidase A enzyme (AGAL). This enzyme is crucial for the degradation of GSLs, and its deficiency leads to the accumulation of globotriaosylceramide (Gb3) in lysosomes. This accumulation affects multiple organs, including the kidneys, heart, and blood vessels, causing symptoms such as renal insufficiency, cardiomyopathy, strokes, and gastrointestinal pain. FD affects 1 in 40,000 to 117,000 people, but it is likely underdiagnosed [80]. Over 1000 mutations in GLA have been identified, with disease severity influenced by environmental factors and blood type; individuals with AB and B blood types often experience more severe symptoms due to additional GSL accumulation in erythrocyte membranes [81]. FD presents differently in males and females, with females often developing symptoms later due to X-chromosome inactivation, which creates a mosaic of normal and mutant cells. Diagnosis in females often requires genetic analysis, as AGAL activity can appear normal [82].

Cardiac symptoms are reported in 40–60% of FD patients, especially those with late-onset FD. The accumulation of Gb3 in FD significantly affects various cardiac structures, including myocytes, intramyocardial vessels, the endocardium, valvular fibroblasts, and conduction tissue. For example, endomyocardial GSL deposition causes enlarged myocytes, eventually making the ventricular walls more rigid and impeding ventricular filling. Ultimately, patients with FD suffer from left ventricular hypertrophy, which may progress to heart failure with preserved ejection fraction [83].

Impaired mitochondrial function and disrupted energy metabolism may also underlie the development of cardiac symptoms in patients with FD. Cultured fibroblasts obtained from FD patients showed significant impairment of mitochondrial function, leading to reduced energy metabolism. Additionally, compared to healthy controls, FD patients demonstrated reduced phosphocreatine and ATP concentrations [84]. Gb3 accumulation has also been implicated in dysregulated autophagy, which can lead to cell death and disease progression. In a human podocyte model of FD, the accumulation of intracellular Gb3 was accompanied by an increased abundance of LC3-II, a marker of autophagosomes, and reduced activity of the mTOR kinase, an inhibitor of autophagic activity [85]. A similar mechanism in cardiomyocytes could contribute to the onset of cardiovascular disease seen in FD patients. Electrocardiographic abnormalities have been shown in FD cardiomyocytes that have an overabundance of Gb3. Compared to control cardiomyocytes, these FD cardiomyocytes showed increased excitability and altered calcium handling [86].

Inflammation also plays a critical role in the progression of FD. Elevated Gb3 levels have been shown to increase apoptotic states in peripheral blood mononuclear cells from FD patients and elevate proinflammatory cytokine expression and production [87,88]. FD patients exhibit higher levels of inflammatory biomarkers such as TNF, IL-6, TNFR1, and TNFR2, which are correlated with deteriorating cardiac function [89]. These findings suggest that glycosphingolipid-induced inflammatory responses significantly contribute to cardiomyopathy in FD.

Understanding the multifaceted impact of Gb3 accumulation on cardiac tissues and the role of inflammation in FD can provide valuable insights into the pathophysiology of cardiac complications and potential therapeutic targets for the management of FD.

### 4.5. Gaucher’s Disease

Gaucher’s disease is another lysosomal storage disorder resulting from deficiency in lysosomal glucocerebrosidase (GCase, also called glucosylceramidase). There are three types of this disease. Type 1 is non-neuronopathic and the most common of the three, affecting about 1 in 40,000 people. Type 2 is acutely neuronopathic, and type 3 is subacutely neuronopathic, occurring in fewer than 1 in 100,000 people [90].

GCase catalyzes the cleavage of glucocerebroside into glucose and ceramide in the intralysosomal membrane. Mutations in the glucocerebrosidase gene (GBA) lead to misfolding of the protein in the ER, disrupting protein trafficking to the lysosomes and ultimately inhibiting GCase activity. Deficiency in GCase activity leads to glucocerebroside accumulation, affecting many organs and organ systems, including the spleen and liver, as well as the skeletal, neurologic, immune, and hematologic systems. Since glucocerebroside is a precursor to many complex GSLs, the absence of functional GCase disrupts a crucial step of sphingolipid metabolism. In GD, glucocerebroside is deacetylated to form glucosylsphingosine (lyso-Gl-1), which accumulates in the lysosomes, causing pH increases and lysosomal destabilization. The dysfunctional lysosomes begin to amass in the cells, interfering with cellular pathways [91]. The deposition of glucocerebroside within these cells (primarily macrophages) results in the appearance of Gaucher cells, which are abnormal cells with small, displaced nuclei and a wrinkled or striated cytoplasm [92].

Cardiac involvement in Gaucher’s disease generally manifests as valvular calcification. The cardiovascular type of GD is GD Type 3c (GD3c), resulting from a rare homozygosity to the p.Asp448His (D409H) GBA mutation. It is primarily the ascending aorta and aortic and mitral valves that are affected, causing stenosis and, eventually, heart failure [93]. Pericarditis and intramyocardial infiltration by Gaucher cells can also occur, leading to constriction and cardiomyopathy [94].

### 4.6. Niemann-Pick Disease

Niemann–Pick disease refers to a group of lysosomal storage disorders that is separated into two subcategories. Niemann–Pick disease types A and B result from mutations in the SMPD1 gene, leading to acid sphingomyelinase deficiency and consequent accumulation of sphingomyelin. Niemann–Pick disease type C results from mutations to either the NPC1 or, rarely, the NPC2 gene, leading to the accumulation of cholesterol, as well as gangliosides and other GSLs, in endosomal compartments. Clinical manifestations of Niemann–Pick disease include liver failure, pulmonary disorders, neurological deficits, and psychiatric symptoms [95]. This review focuses on Niemann–Pick disease type C (NPC).

The full functions of NPC1 and NPC2 have yet to be elucidated. NPC1 is localized to vesicles involved in the recycling of unesterified cholesterol from late endosomes/lysosomes to the ER and Golgi and may also function in GSL homeostasis [96]. Therefore, disruptions in NPC1 activity disrupt the transfer of cholesterol and other lipids, causing lower-than-normal amounts of these lipids to reach the plasma membrane and ER and an accumulation of lipids in multiple tissues. Indeed, tissue extracted from mice with NPC1 knockout was highly enriched in GSLs, sphingosine, and cholesterol compared to normal mice. Furthermore, NPC cell culture models showed altered endocytic trafficking and decreased fluid-phase uptake, which was reversed by GSL-lowering drugs and the inhibition of GSL synthesis [97]. Similarly, murine and feline models of NPC treated with NB-DNJ, a GSL synthesis inhibitor, showed reduced ganglioside accumulation and ameliorated neurological disease symptoms [96]. These results suggest a pivotal role of GSLs and GSL synthesis in NPC disease.

In the cardiovascular system, NPC1 has been linked to atherosclerosis. Apoe−/− mice, the standard mouse model for atherosclerosis, were crossed with Npc1−/− mice to create a double mutant. These mice showed greater atherosclerotic lesion areas than their Apoe−/− littermates and were also at greater risk of atherothrombosis and medial degradation, acute complications in patients with atherosclerosis [98]. On the other hand, NPC1 heterozygosity in mice conferred protection against lesional necrosis and subsequent atherosclerotic plaque instability compared to NPC1+/+ mice [99]. The role of NPC1 in the cardiovascular system is further supported by cohort studies linking common genetic variants associated with NPC with cardiovascular complications. For example, Afzali et al. showed that methylation of the NPC1 promoter was a risk factor for cardiovascular disease (CVD) and that levels of total triglycerides, cholesterol, HDL-C, and LDL-C vary with the methylation level, with the unmethylated promoter correlating with lower levels than the methylated promoter [100]. Similarly, Ma et al. showed a correlation between NPC1 variants and coronary heart disease (CHD) [101]. Finally, Zhao et al. showed a relationship between an indel polymorphism downstream of NPC1 (rs150703258) and sudden cardiac death (SCD) [37]. However, these studies are limited by a relatively small cohort size and low ethnic diversity.

## 5. Clinical Studies on Glycosphingolipids in Cardiovascular Disorders

Clinical studies exploring glycosphingolipids (GSLs) in cardiovascular disorders primarily focus on biomarker identification for high cardiovascular risk. However, emerging research has begun to unravel the mechanistic roles of GSLs in acute cardiovascular events. For instance, a pivotal study by Knapp et al. investigated alterations in sphingolipid plasma concentrations following acute ST-segment elevation myocardial infarction (STEMI) [102]. The study revealed that patients with STEMI exhibited significantly reduced plasma levels of sphingoid base-1 phosphates such as S1P and sphinganine-1 phosphate (SA1P) [102], suggesting a disruption in sphingolipid-mediated signaling pathways that are crucial for cardiac protection.

Furthermore, erythrocytes from STEMI patients showed transient increases in sphingoid bases and sphingolipids [102], suggesting a compensatory response to the acute reduction in plasma levels. This response may involve altered sphingolipid metabolism within erythrocytes or a shift in their release or degradation dynamics, possibly mediated by vascular endothelium interactions. The persistent discrepancy in plasma and erythrocyte sphingolipid levels post STEMI underscores the complex regulatory mechanisms governing cardiac sphingolipid homeostasis.

Moreover, the impact of standard antiplatelet therapies on sphingolipid profiles complicates the interpretation of these differential distributions of sphingolipids. Subsequent research by Knapp et al. evaluated the influence of aspirin on sphingolipid concentrations across different blood compartments [102]. Results indicated that aspirin administration could modulate sphingolipid metabolism, affecting the levels of cardioprotective sphingoid base-1 phosphates in the bloodstream [102].

These studies collectively highlight the critical role of GSLs and their metabolic pathways in modulating cardiac function and response to ischemic injury [102]. Understanding these pathways offers potential therapeutic avenues for the enhancement of cardioprotection and the tailoring of interventions to manage and prevent the progression of heart failure.

## 6. Glycosphingolipids as Biomarkers in Cardiovascular Diseases

The compound that has been most consistently studied as an indicator of cardiovascular disease is ceramide. Many studies have suggested that disturbances in the balance between ceramide and sphingosine-1 phosphate may move forward the induction of apoptosis [56], underscoring why it is so important to examine them as biomarkers.

Spijkers et al. investigated plasma ceramide levels in patients diagnosed with Stage 2 and Stage 3 essential hypertension and found that ceramide levels were significantly higher than in normotensive controls. Even more interestingly, ceramide levels were correlated with severity of disease, with increases in C24:1 and C24:0 ceramides accounting for the majority of the increase in plasma ceramide concentrations. They did not find any significant changes in sphingomyelin or ceramide-1 phosphate (C1P) levels, although C1P did trend downwards for hypertensive patients [103].

Delving deeper into the different forms of ceramides at play in vascular health, a recent study evaluated the effect of a Mediterranean diet on various indices, including lipid and ceramide serum concentrations, through a randomized controlled trial. The intervention of the Mediterranean diet was intended to mitigate the effects of higher ceramide concentrations on cardiovascular disease risk. At six months, subjects in the intervention arm showed increased serum concentrations of Cer-24 and decreased serum concentrations of Cer-22 and the Cer-24/Cer-16 ratio. At 12 months, they showed even higher serum concentrations of the Cer-24/Cer-16 ratio and lower serum concentrations of C24:0 and C18. The study indicated that an increase in the Cer-24/Cer-16 ratio is inversely related to cardiovascular risk, suggesting a potential cardioprotective role [104]. Similarly, a meta-analysis investigated the association between specific ceramide species and cardiovascular disease, finding that major adverse cardiovascular events (MACEs) were associated with elevated plasma concentrations of Cer (d18:1/16:0), Cer (d18:1/18:0), and Cer (d18:1/24:1), whereas levels of Cer (d18:1/22:0) and Cer (d18:1/24:0) were not similarly increased [105]. In the PREDIMED trial, patients were randomized to different variations of the Mediterranean diet. Researchers calculated a “ceramide score” for each participant based on a weighted sum of plasma concentrations of C16:0, C22:0, C24:0, and C24:1. They discovered that a higher ceramide score was associated with more than double the risk of cardiovascular disease (CVD), including non-fatal acute myocardial infarction, non-fatal stroke, and cardiovascular death. This finding suggests that these specific plasma ceramide concentrations are independently correlated with an increased risk of CVD (non-fatal acute myocardial infarction, non-fatal stroke, or cardiovascular death) [106] and that the ratios of ceramide species were not more strongly associated with CVD risk than individual ceramides, which is notably different than the evidence put forth by Daidone et al. [104].

The idea that total ceramide levels are more relevant than ceramide composition in predicting CVD risk has become less prominent over the years, as recent studies like that conducted by Yin et al. [107] have demonstrated the utility of factoring in specific species ratios to evaluate risk. Yin et al. [107] proposed that different populations may have different relevant combinations of ceramides to examine, and they chose to find a good fit for hypertensive patients at high CVD risk as compared to ceramide score 1 (CERT-1), which is a conventional scoring system based on plasma concentrations of specific ceramides that groups patients into one of four risk groups. The novel ceramide score for hypertensive patients (CERT-HBP) significantly improved the predictive value of the measured plasma concentrations of ceramides. CERT-HBP consisted of Cer (d18:1/16:0) and its ratio to Cer (d18:1/22:0), as well as Cer (d18:1/24:1) and its ratio to Cer (d18:1/24:0), as this species had previously been proven to be associated with CVD risk [107]. In contrast, the CERT-2 score employed by Hilvo et al. [108] to detect residual risk in patients with stable CAD comprises three lipid ratios (Cer (d18:1/24:1)/Cer (d18:1/24:0), Cer (d18:1/18:0)/phosphatidylcholine 14:0/22:6, and Cer (d18:1/16:0)/phosphatidylcholine 16:0/22:5) and a single lipid (phosphatidylcholine 16:0/16:0) [108]. This particular metric proved useful in evaluating residual risk in stable CAD patients, just as the prediction of MACE in hypertensive patients based on the CERT-HBP metric was also significantly improved. Other sphingolipids of note for positive associations with CAD include sphingosine, dihydro-Cer (d18:0/16:0), dihydro-Cer (d18:0/18:1), dihydro-SM (d18:0/24:1), dihydro-SM (d18:0/22:0), SM (d18:1/18:0), Cer (d18:1/18:0), and Cer (d18:1/24:0), all of which were identified by unbiased machine learning conducted on targeted serum lipidomics [103]. This underscores the suggestion that different combinations of ceramides may be necessary to assess a variety of CVD risks in different patient populations.

Interestingly, Saleem et al. examined the roles of C22:0 and C24:0 in pathological neurodegeneration associated with coronary artery disease (CAD) by assessing them as predictors of verbal memory performance in CAD patients. High concentrations of both ceramide species were found to be significantly predictive of less verbal memory performance improvement over one year, adding a new dimension to the use of ceramides as a biomarker of disease [109].

In addition to ceramide, sphingomyelin (SM) and secretory acid sphingomyelinase (S-SMase) have been investigated as potential biomarkers. When comparing plasma concentrations of all three compounds between healthy controls, patients with stable angina pectoris (SAP), patients with unstable angina pectoris (UAP), and patients with acute myocardial infarction (AMI), ceramide and S-SMase levels were highest in the AMI and UAP groups. Surprisingly, S-SMase activity was higher in the UAP group than in the AMI group [110].

Recent studies, including a significant analysis of the PREDIMED and EPIC-Potsdam cohorts, have shown that certain sphingolipid species, particularly Cer C16:0, are not only increased in individuals with heart failure but also serve as top lipid cluster networks associated with the condition [111]. These findings suggest a deeper interconnection between sphingolipid profiles and heart failure pathogenesis, expanding our understanding of how these biomarkers could potentially influence diagnostic and therapeutic strategies in cardiac care.

## 7. Therapeutic Potential of Targeting Glycosphingolipids

### 7.1. Enzyme Replacement Therapy (ERT)

In diseases caused by a deficiency of enzyme activity, resulting in a build-up of GSLs, enzyme replacement therapy (ERT) may compensate for the lack of normal enzymatic activity and provide a viable treatment option. Gaucher’s disease, which is characterized by a deficiency in glucocerebrosidase (the enzyme that hydrolyzes the glucose moiety from glucosylceramide) is one such disease. ERT for GD was introduced in 1991. There are three available ERTs for GD, namely imiglucerase (a recombinant GCase produced from Chinese hamster ovaries), velaglucerase alfa (a gene-activated GCase produced from human fibroblasts), and taliglucerase alfa (produced from carrot cells). In long-term observational studies, patients treated with these ERTs have shown improvements that were sustained for 20 years with continued treatment. These results were consistent, regardless of pre-treatment disease severity and were accompanied by limited side effects [112,113,114]. However, due to the low stability of the enzyme in blood, ERT is a lifelong treatment requiring periodic intravenous administration, leading to extremely high costs. In addition, due to the impermeability of the blood–brain barrier, ERT is less successful in treating the neurologic symptoms of GD. These drawbacks have led to the use of nanotechnology for improved delivery of ERT [115,116].

Similarly, Fabry’s disease, which is characterized by a deficiency in α-galactosidase A activity, causing a buildup of globotriaosylceramide (Gb3), can be treated with ERT. Agalsidase beta and agalsidase alfa have been commercially available since 2003, and treated patients show improved outcomes [117,118]. Pegunigalsidase alfa, a novel, PEGylated, chemically modified α-Gal A enzyme with covalently crosslinked monomers, was also recently approved to treat FD [119]. It has a plasma half-life of about 80 h, compared to about 2 h for the other two ERTs, and shows reduced immunogenicity [119].

### 7.2. Substrate Reduction Therapy (SRT)

Another strategy for treating diseases caused by an accumulation of GSLs is substrate reduction therapy (SRT), which involves decreasing the biosynthesis of the accumulated GSL. Miglustat acts as a competitive inhibitor of glucosylceramide synthase, decreasing the synthesis and accumulation of glucosylceramide in GD. However, adverse events such as gastrointestinal disturbance have been reported in some patients [120]. Eligustat is also approved for GD patients and has proven to be equivalent to ERT and less toxic than miglustat, making it the frontline treatment of choice for patients with GD1 [121]. For patients with FD, venglustat and lucerastat (the galactose form of miglustat) are two SRTs that are being tested [122]. SRTs may be preferable to ERTs due to the relative ease of administration (oral vs. intravenous), the ability of some to cross the BBB, and the reduced likelihood of inducing anti-drug antibodies (ADAs) [122].

In addition, glucosylceramide synthase inhibitors (GCSis) have been studied in mice for the treatment of cardiac hypertrophy. As mentioned above, D-PDMP, an inhibitor of glucosylceramide synthase and lactosylceramide synthase activity, decreased GSL load and inhibited cardiac hypertrophy in a dose-dependent manner [16,17]. Similarly, Baccam et al. demonstrated that GZ667161, a small-molecule GCSi, protects mice against isoproterenol- and chronic kidney disease-induced cardiac dysfunction in mice [123]. Other GCSis, like Genz-123346, have been studied for their efficacy in arresting the growth of tumors, in which glucosylceramide synthase is commonly overexpressed [124]. These studies, in combination with the proven safety and efficacy of GCSis in treating lysosomal storage disorders through SRT, suggest that a similar strategy can be utilized to treat cardiac hypertrophy.

### 7.3. Chaperone-Mediated Therapy (CMT) or Pharmacological Chaperone Therapy (PCT)

Chaperone mediated therapy (CMT) uses pharmacologically active molecules to recover enzyme activity by correcting misfolding and enhancing the enzyme’s stability. In Gaucher’s disease, misfolded GCase is degraded in the ER, leading to decreased enzymatic activity. Several chaperones are used to treat GD, including ambroxol and N-Octyl-b-valienamine [125]. Similarly, for Fabry’s disease, migalastat is used to bind and stabilize mutant α-galactosidase A, increasing its activity and improving lysosomal trafficking [126]. These treatments result in reduced GSL load, leading to improved health outcomes. However, because CMT is mutation-specific, results can vary from patient to patient, illustrating the need for personalized medicine approaches in these therapies [125].

## 8. Methodologies for Determining GSLs

Recent advancements in glycan analysis techniques have significantly deepened our understanding of GSLs and their complex roles across various health and disease states. Mass spectrometry techniques, including MALDI-TOF MS and ESI-MS, have become pivotal in identifying and quantifying GSLs, allowing for detailed characterization of their molecular structures [127]. This level of analysis is crucial for exploring how specific GSL structures influence cellular functions and disease mechanisms. High-performance liquid chromatography (HPLC), often coupled with fluorescence detection or mass spectrometry, provides robust profiling of GSLs based on carbohydrate composition, which is invaluable in studies on cancer and neurological disorders where GSLs play key roles [128]. Furthermore, nuclear magnetic resonance (NMR) spectroscopy offers insights into the three-dimensional structures of GSLs, showing how these molecules interact within cell membranes to affect signaling, immune responses, and cellular adhesion [128]. Additionally, the emergence of glycan microarray technology allows for the screening of GSL–protein interactions, highlighting GSLs’ roles in immune modulation and pathogen recognition [128].

## 9. Conclusion and Future Directions

Our comprehension of glycosphingolipids (GSLs) within cardiovascular contexts is presently constricted and mainly derived from explorations that emphasize GSLs’ roles in cellular signaling and structural support. The molecular complexity of GSLs has historically impeded detailed studies, a challenge further exacerbated by limitations in the available analytical techniques. Recent technological advancements in glycan analysis, such as MALDI-TOF MS and ESI-MS, have significantly enhanced our capacity to identify and quantify GSLs, facilitating more comprehensive investigations into their diverse roles in health and disease. While emerging data offer some insights into heart failure, extensive mechanistic studies are essential, considering much of the current literature predominantly addresses correlations without establishing causality. As research progresses, the integration of GSL metabolic pathways into the broader framework of cardiovascular disease management promises not only to refine therapeutic strategies but also to improve outcomes for patients with complex cardiovascular conditions. This continued exploration is poised to unveil pivotal insights into the systemic impacts of GSLs, potentially driving the development of personalized medicine approaches that tailor treatment efficacy based on specific GSL profiles.

## Figures and Tables

**Table 1:** GSL classification according to core structures.

Series	Abbreviation	Core Structure
Ganglio-	Gg	Galb3GalNAcb4Galb4Glc-
Globo-	Gb	GalNAcb3Gala4Galb4Glc-
Isoglobo-	iGB	GalNAcb3Gala3Galb4Glc-
Lacto-	Lc	Galb3GlcNAcb3Galb4Glc-
Neolacto-	nLc	Galb4GlcNAcb3Galb4Glc-
Mollu-	Mu	GlcNAcb2Mana3Manb4Glc-
Arthro-	At	GalNAcb4GlcNAcb3Manb4Glc-

## Data Availability

Not applicable.
